# Pomegranate Metabolites Impact Tryptophan Metabolism in Humans and Mice

**DOI:** 10.1093/cdn/nzaa165

**Published:** 2020-11-25

**Authors:** Jieping Yang, Yuanqiang Guo, Rupo Lee, Susanne M Henning, Jing Wang, Yajing Pan, Tianyu Qing, Mark Hsu, Alex Nguyen, Siddarth Prabha, Rashi Ojha, Gary W Small, David Heber, Zhaoping Li

**Affiliations:** Center for Human Nutrition, David Geffen School of Medicine at UCLA, Los Angeles, CA, USA; State Key Laboratory of Medicinal Chemical Biology, College of Pharmacy, and Tianjin Key Laboratory of Molecular Drug Research, Nankai University, Tianjin, China; Center for Human Nutrition, David Geffen School of Medicine at UCLA, Los Angeles, CA, USA; Center for Human Nutrition, David Geffen School of Medicine at UCLA, Los Angeles, CA, USA; Center for Human Nutrition, David Geffen School of Medicine at UCLA, Los Angeles, CA, USA; Key Laboratory of Health Cultivation of the Ministry of Education, Beijing University of Chinese Medicine , Beijing, China; Key Laboratory of Health Cultivation of the Ministry of Education, Beijing University of Chinese Medicine , Beijing, China; Center for Human Nutrition, David Geffen School of Medicine at UCLA, Los Angeles, CA, USA; Center for Human Nutrition, David Geffen School of Medicine at UCLA, Los Angeles, CA, USA; Department of Psychiatry and Biobehavioral Sciences, Semel Institute for Neuroscience and Human Behavior, David Geffen School of Medicine at UCLA, Los Angeles, CA, USA; Department of Psychiatry and Biobehavioral Sciences, Semel Institute for Neuroscience and Human Behavior, David Geffen School of Medicine at UCLA, Los Angeles, CA, USA; Department of Psychiatry and Biobehavioral Sciences, Semel Institute for Neuroscience and Human Behavior, David Geffen School of Medicine at UCLA, Los Angeles, CA, USA; Center for Human Nutrition, David Geffen School of Medicine at UCLA, Los Angeles, CA, USA; Center for Human Nutrition, David Geffen School of Medicine at UCLA, Los Angeles, CA, USA; Department of Medicine, VA Greater Los Angeles Health Care System , Los Angeles, CA, USA

**Keywords:** ellagic acid, urolithin A, pomegranate juice, tryptophan metabolism, gut microbiota

## Abstract

**Background:**

We showed that pomegranate juice (PomJ) can help to maintain memory in adults aged >50 y. The mechanism for this effect is unknown, but might involve Trp and its metabolites, which are important in brain function.

**Objectives:**

We aimed to test the hypothesis that PomJ and its metabolites ellagic acid (EA) and urolithin A (UA) affect Trp metabolism.

**Methods:**

Stool and plasma from a cohort [11 PomJ, 9 placebo drink (PL)] of subjects enrolled in our double-blind, placebo-controlled trial (NCT02093130) were collected at baseline and after 1 y of PomJ or PL consumption. In a mouse study, cecum and serum were collected from DBA/2J mice receiving 8 wk of dietary 0.1% EA or UA supplementation. Trp metabolites and intestinal microbiota were analyzed by LC-MS and 16S rRNA gene sequencing, respectively.

**Results:**

In the human study, the change in the plasma Trp metabolite indole propionate (IPA) over 1 y was significantly different between PomJ and PL groups (*P *= 0.03). In serum of experimental mice, we observed a 230% increase of IPA by EA but not UA, a 54% increase of indole sulfate by UA but not EA, and 43% and 34% decreases of kynurenine (KYN) by EA and UA, respectively. In cecum, there was a 32% decrease of Trp by UA but not EA, and an 86% decrease of KYN by EA but not UA (*P *< 0.05). The abundance of 2 genera, *Shigella* and *Catenibacterium*, was reduced by PomJ in humans as well as by UA in mice, and their abundance was negatively associated with blood IPA in humans and mice (*P *< 0.05).

**Conclusions:**

These results suggest a novel mechanism involving the regulation of host and microbial Trp metabolism that might contribute to the health benefits of ellagitannins and EA-enriched food, such as PomJ.

## Introduction

Trp is an essential amino acid, and Trp metabolism has been involved in many aspects of host metabolism and physiology (1, 2). Both host and gut microbiota are involved in Trp metabolism. The majority of Trp is metabolized through the kynurenine (KYN) pathway by host cells, which generates many bioactive metabolites important for immune regulation (3). Trp is also metabolized into many neuroactive metabolites, such as serotonin by host cells, and indole derivatives by gut microbiota (3, 4). Although host cells catabolize Trp into serotonin, indigenous spore-forming bacteria from the gut microbiota promote serotonin biosynthesis in colonic enterochromaffin cells by regulating other bacterial metabolism (4). Serotonin and indole derivatives, such as indole propionate (IPA), also have a regulatory role in immune responses as well as host metabolism (5–8).

We previously reported that daily consumption of 237mL of pomegranate juice (PomJ, *n* = 15) for 1 mo improved memory performance and altered brain neural activity in older subjects with mild memory complaints compared with daily intake of taste-matched placebo juice (PL) with high fructose content but no polyphenols (*n* = 13) (9). In addition, we recently evaluated the effects of long-term PomJ consumption on cognitive ability of healthy middle-aged and older adults. We found that subjects in the PomJ group who consumed 237mL of PomJ daily for 12 mo (*n* = 98) experienced stabilization of performance of a memory score involving visual-spatial learning compared with subjects in the PL group (*n* = 102) who consumed the PL and showed a decline on that learning score (10). The underlying mechanism of improvement of memory performance and metabolic markers associated with PomJ consumption remains largely unknown (9–12). PomJ contains a variety of bioactive compounds such as phenolics [ellagitannins (ETs) and ellagic acd (EA)] and flavonoids (anthocyanins, etc.) (13, 14). After oral consumption, ETs are hydrolyzed to EA in the intestine. EA can be absorbed into the bloodstream, or remain in the intestine to be further transformed into urolithins, such as urolithin A (UA), by gut microbiota. ETs and EA are poorly bioavailable, but UA has 10-fold better bioavailability compared with EA (15–18). The beneficial effects of ETs, EA, and their microbial metabolite UA have been reported (17, 19–21).

PomJ intake was found to acutely regulate the concentrations of the Trp metabolite melatonin in both healthy and insulin-resistant (IR) subjects (11). EA supplementation was associated with neuroprotective, analgesic, antiamyloidogenic, and antidiabetic effects (22–24). Metabolism of Trp into serotonin was found to be critical for the antidepressant-like activity of EA (25). We therefore hypothesized that PomJ and its bioactive component EA likely induce alterations of the microbial and host metabolism of Trp, which could be a potential mechanistic link underlying the observed health benefits of PomJ. In humans, ∼25–80% of study subjects produce UA from ETs/EA after consuming ETs/EA-containing food (26, 27). Individuals differ in their ability to convert ETs/EA to UA after ETs/EA intake, resulting in large interindividual differences in the concentrations of EA and UA present in the gut and circulation (26, 27). Results from previous studies suggest that EA and UA share certain similar biological activities, but also have distinct functions (20, 28, 29). The health benefits of UA in aging and inflammatory bowel disease (IBD) are of great interest. A recent clinical trial has reported improved mitochondrial and cellular health in humans supplemented with UA (30). The aims of this study included: *1*) investigating the possible mechanism of PomJ on memory by evaluating the effect of PomJ intake on Trp metabolism; *2*) investigating the individual effects of EA and UA on Trp metabolism by supplementing EA and UA individually in mice lacking the ability to produce UA; and *3*) investigating the effect of PomJ, EA, and UA intake on gut microbiota and the association with Trp metabolism.

For this purpose, we first analyzed plasma Trp metabolites and fecal microbiota from a small cohort of subjects enrolled in our recent randomized controlled trial comparing memory performance of 1-y PomJ compared with PL consumption in healthy older adults (10). We recently showed the differential metabolic effects of EA and UA in mice with high-fat/high-sucrose (HFHS) diet–induced IR (31). The antidiabetic and neurotropic effects of EA were reported in diabetic rats, and a contribution of Trp metabolism to the EA-mediated neurotropic effects has previously been reported (24, 25). We therefore used mice with HFHS diet–induced IR as a model to study the individual effects of EA and UA on Trp metabolism. However, whether dietary macronutrients affect these effects requires further investigation.

## Methods

### Human study

We recently evaluated the memory performance in subjects consuming 237 mL/d PomJ (*n* = 98) or PL (*n* = 102) for 1 y in a randomized, double-blind, 2-arm, parallel-design study (10). The study was carried out at the Semel Institute for Neuroscience and Human Behavior and Center for Human Nutrition, David Geffen School of Medicine at the University of California, Los Angeles, CA in accordance with the guidelines of the Human Subjects Protection Committee of the University of California, Los Angeles. All subjects gave written informed consent before the study began. This study was registered at clinicaltrials.gov as NCT02093130. Subjects (aged 50–70 y) were randomly allocated to consume 237 mL of PomJ (Wonderful Company, LLC) or PL every day. The PL contained matched constituents as PomJ (37g sugar from high-fructose corn syrup, flavor and acidity level) except for phenolic compounds (3). A subset (11 PomJ and 9 PL) of 20 subjects who provided stool and blood samples at baseline and final visit were selected for the analysis. Stool samples were used for microbiota analysis. Plasma samples were used for Trp metabolite analysis. Data from 1 subject from the PL group were excluded due to detection of Pom metabolites in the blood. Data about the cognitive outcomes from this clinical trial were published previously (3).

### Animal study

All mouse procedures were approved by the UCLA Animal Research Committee in compliance with the Association for Assessment and Accreditation of Laboratory Care International. Twenty-four male DBA/2J mice aged 5–6 wk were purchased from the Jackson Laboratory. After 1 wk of acclimation, PomJ was used to replace drinking water for 4 d, and stool samples were collected every day for 4 d to confirm the lack of UA production capability. Mice were then switched to regular water and fed an HFHS diet (42% energy from fat, 30% energy from sucrose) for 8 wk; they were then randomly assigned to 1 of 3 groups, and fed either an HFHS diet, or an HFHS diets supplemented with 0.1% EA (94% EA; Ecological Formulas), or 0.1% UA (97% UA; Feitang) (**Supplemental Table 1**). Two hundred and thirty-seven millilitres PomJ provides ∼120mg EA when completely hydrolyzed (32). EA and UA were supplemented at 0.1% to HFHS-fed mice in this study, which is similar to a daily intake of 780 mg in humans. This dose is about 7-fold higher than a daily consumption of 237 mL of PomJ in humans (31). However, the mouse study was a short-term experiment compared with the 1-y human study, and it is has been shown that humans can tolerate EA and UA at 500–1000 mg/d (16.7 mg/kg) via oral administration (30, 32). After EA or UA feeding for 8 wk, mice were killed, and blood and cecum contents were collected, weighed, and stored at −80°C until analysis (33).

### Measurement of Trp and its major metabolites

Fifty microliters of human plasma or mouse serum samples were precipitated with 500 µL methanol and centrifuged at 10,000 × *g* for 10 min at 4°C. Supernatant was dried using a SpeedVac evaporator (ThermoFisher Scientific) and then resuspended in 50% methanol for analysis. LC coupled to electrospray ionization triple quadrupole MS (LC-ESI-MS/MS) at positive mode was used to determine Trp, serotonin, tryptamine, kynurenic acid (KYNA), and KYN, and HPLC fluorescence was used to determine indole acetate (IAA), indole sulfate (IS), and IPA at excitation/emission 280nm/370nm. The LC-ESI-MS/MS was performed on a 2.1 × 150-mm Agilent Zorbax C18 column. The mobile phase for LC-ESI-MS/MS analysis consisted of 2 solutions: acetonitrile and 0.25% formic acid/H_2_O. Tandem MS spectra were automatically performed with argon as the collision gas (Trp: *m/z* 205/146; serotonin: *m/z* 177/160; KYN: *m/z* 209/146; tryptamine: *m/z* 161/144; and KYNA: *m/z* 190/144). The HPLC was performed on an Inertsil ODS-4 4.6 × 150-mm column. The mobile phase for HPLC analysis consisted of 2 solutions: acetonitrile and 0.2% trifluoroacetic acid/H_2_O. Concentrations were calculated by comparing sample peak area with the commercial standard peak area. For every 10 samples, we randomly picked 1 and spiked that with known concentrations of mixed standards in duplicate to evaluate the accuracy and precision of the LC-ESI-MS/MS and HPLC methods. We used the following equation to calculate percentage recovery:

% recovery = 100 × (measure value for spiked samples − measure value for unspiked samples)/known value of mixed standards

Samples with the percentage of recovery between 80% and 120% were included in the results.

### Sample preparation for blood amino acid HPLC analysis

Twenty-five microliters human plasma or mouse serum samples were mixed with 500 µL of methanol, vortexed for 1 min and then centrifuged at 10,000 × *g* for 10 min at 4°C. Supernatant was dried by SpeedVac and reconstituted in 10 µL water. Seventy microliters AccQ Fluor Borate buffer and 20 µL reconstituted AccQ Fluor Reagent were added to the sample tubes following manufacturer's manual (AccQ Fluor Reagent kit, Waters Corp.). Samples were vortexed for 1–2 min and incubated at 55°C for 10 min. Samples were then cooled at room temperature and centrifuged, and the supernatant was used for HPLC analysis as described above.

### 16S rRNA gene sequencing and taxonomic analysis

DNA from stool or cecum content was extracted using the DNeasy PowerSoil Kit (Qiagen). Sequencing of the V4 variable region was performed at MR DNA (www.mrdnalab.com) on a MiSeq sequencer (Illumina). Sequence data were processed using the MR DNA analysis pipeline as previously described (34). Final operational taxonomic units were taxonomically classified using BLASTn against a curated database derived from Greengenes 12_10 (35), RDPII (http://rdp.cme.msu.edu), and the National Center for Biotechnology Information (www.ncbi.nlm.nih.gov) as previously described (34). All taxonomic analyses were conducted in R (version 3.5.2; R Foundation) (36) with phyloseq (37), ggplot2 (38), vegan (39), and DESeq2 (40) packages as previously described (34). α-Diversity indexes (Chao1 and Shannon) were estimated using count value after rarefication. Measure of β-diversity was performed using Bray–Curtis dissimilarity. The relations of samples across groups were determined by permutational multivariate analysis of variance using the Adonis command provided by vegan in R and were displayed via principal coordinate analysis (PCoA) ordination.

DESeq2 was used to identify abundance changes at the genus level that occurred differentially between PomJ and PL groups. Null and test models were constructed, and an interaction between “time (baseline and final)” and “intervention (PL or PomJ)” was the differentiating term between 2 models as previously described (34). A likelihood ratio test was used to identify differentially abundant genera between PL and PomJ groups. Negative binomial Wald test provided in DESeq2 was used to identify genera of differential abundance between groups or between baseline and final within group as previously described (34). *P* values were adjusted for multiple testing using the Benjamini–Hochberg false discovery rate correction in DESeq2. Because Bonferroni correction is often considered overly conservative, we listed genera with *P* values < 0.05 and marked those with adjusted *P* < 0.2 using an asterisk (*).

### Statistical analysis

Statistical analysis was performed using the Statistical Package for the Social Sciences version 8.0 software (SPSS Inc.). Summary statistics (mean, SD, and SEM) were calculated. The sample size was determined based on a previous study showing that 200 mL PomJ intake for 4 wk effectively changed phenolic metabolites and gut bacteria (*n* = 12) (41). For the human intervention study, Mann–Whitney test and Fisher exact test were used to analyze differences in baseline characteristics between groups, and Wilcoxon signed rank test was used to analyze the differences in measures (final − baseline) within groups. The Mann–Whitney test was also used to see if the changes in dependent variables in the 2 groups were different, including BMI, body weight, and Trp metabolites. For animal data, 1-factor ANOVA was used when data were normally distributed. The Tukey–Kramer multiple comparison procedure was used for post hoc comparisons. The Kruskal–Wallis test with Bonferroni correction was used when data were not normally distributed. *P* values < 0.05 were considered statistically significant.

## Results

### Effects of PomJ on plasma concentrations of Trp metabolites in humans

**FIGURE 1:**
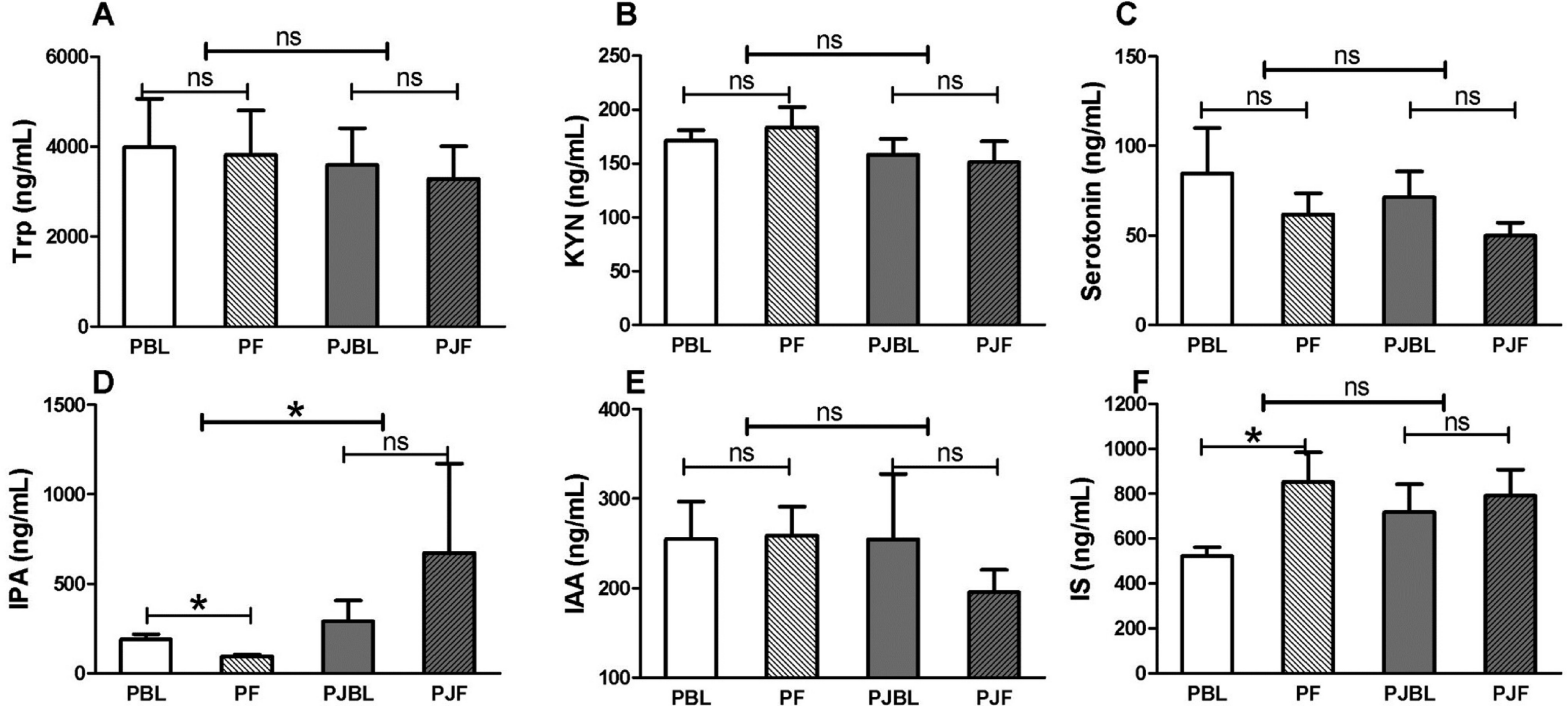
Human plasma Trp and its major metabolites at baseline and after 1 y of PomJ or PL intake. (A) Trp, (B) KYN, (C) serotonin, (D) IPA, (E) IAA, and (F) IS concentrations. Data are presented as means ± SEMs (PomJ, *n* = 11; PL, *n* = 8). Within-group changes were analyzed by Wilcoxon signed ranks test (light capped line; PBL vs. PF; PJBL vs. PJF). Mann–Whitney test was used to see if the changes of Trp metabolites in the 2 groups are different (bold capped line; PL vs. PomJ). *Significantly different, *P* < 0.05. IAA, indole acetate; IPA, indole propionate; IS, indole sulfate; KYN, kynurenine; ns, nonsignficant; PBL, placebo baseline; PF, placebo final; PJBL, PomJ baseline; PJF, PomJ final; PL, placebo; PomJ, pomegranate juice.

Subject characteristics are shown in **Table 1**. There were no differences in baseline demographic characteristics in the PL and PomJ groups. The change in plasma IPA over time in the PL group was significantly different (*P* = 0.03) from that in the PomJ group. In the PL group, IPA concentrations decreased, whereas in the PomJ group the IPA concentrations were stable over 1 y of PomJ consumption (**Figure 1**D). Plasma concentrations of IS increased significantly in the PL group (*P* = 0.008), whereas IS concentrations remained stable over 1 y of PomJ consumption (Figure 1F). Trp and other major Trp metabolites, including serotonin, KYN, and IAA (Figure 1A–C, E) did not change significantly in either group.

**TABLE 1:** Demographics of study participants^1^

	Placebo (PL)	Pomegranate juice (PomJ)	*P*
Sex, % women	75%	82%	0.57
Age, y	57.9 ± 1.8	59.7 ± 4.9	0.56
BMI, kg/m^2^ (baseline)	26.0 ± 4.2	26.9 ± 5.2	0.66
BMI, kg/m^2^ (final)	26.5 ± 4.3	26.1 ± 5.1	0.84
Body weight, kg (baseline)	71.8 ± 15.4	71.5 ± 15.1	0.90
Body weight, kg (final)	73.1 ± 14.9	69.6 ± 15.5	0.60

1 Data are means ± SDs (PomJ, *n* = 11; PL, *n* = 8). Fisher exact test and Mann–Whitney test were used to compare demographic characteristics (sex, age, BMI, and body weight) at baseline between PL and PomJ, respectively. Wilcoxon signed rank test was used to compare the changes in BMI and body weight between final and baseline within groups. Mann–Whitney test was also used to see if changes in BMI and body weight in the 2 groups were different. *P* < 0.05 is significant. No significant differences were detected for any comparisons.

### Effects of PomJ on gut microbiota

**FIGURE 2:**
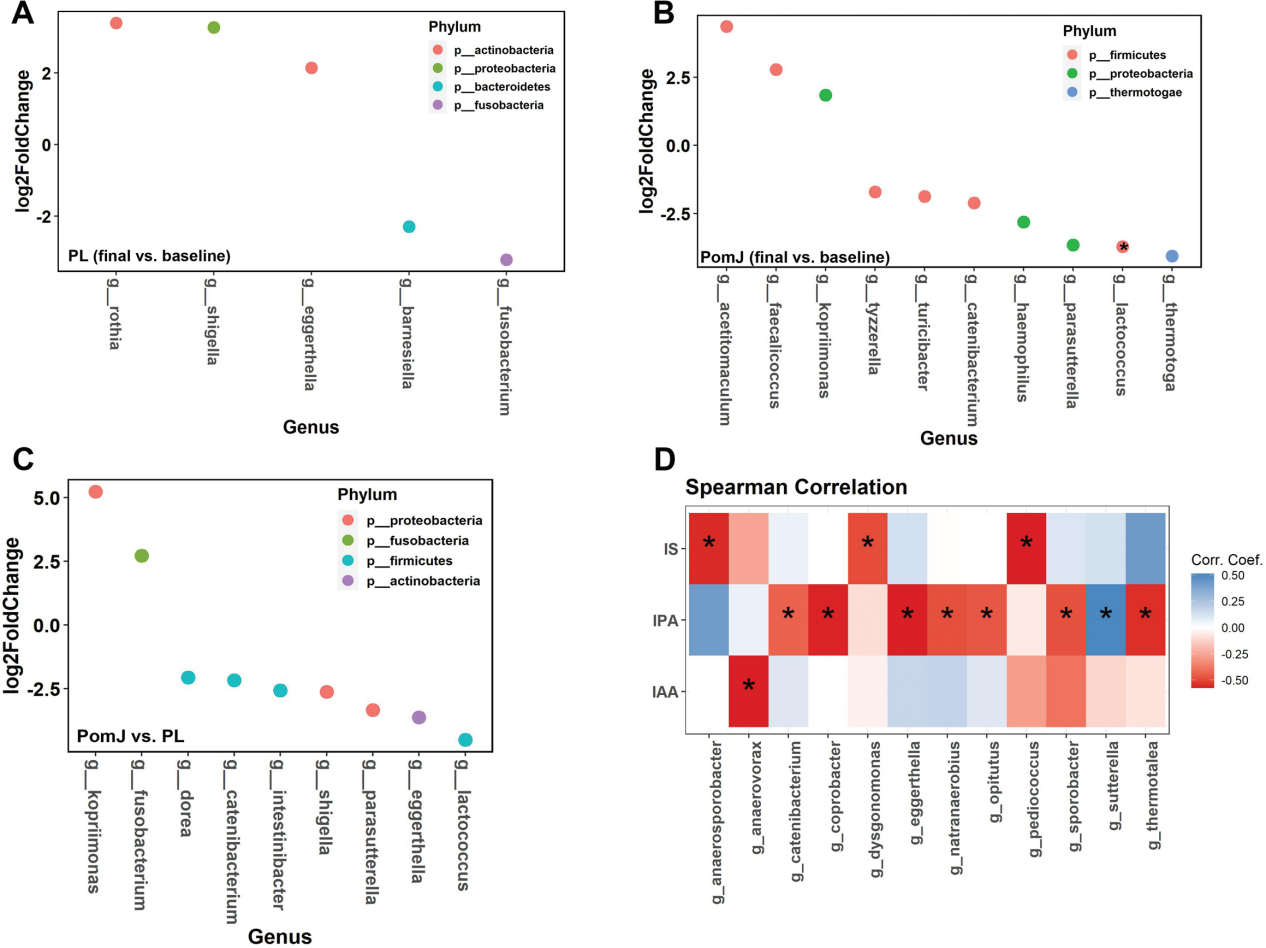
Impact of PomJ and PL drink on gut microbiota. Microbial genera identified to be significantly different in abundance between baseline and final in (A) PL (*n* = 8) and (B) PomJ (*n* = 11) by DESeq2 (40) (*P* < 0.05; *adjusted *P* < 0.2). In DESeq2, negative and positive log_2_-fold change value denotes decreased or increased abundance at final compared with baseline, respectively. (C) Genera identified to be significantly different in abundance by DESeq2 between PomJ and PL, including baseline and final (*P* < 0.05). (D) Changes from final and baseline visits in circulating Trp microbial metabolite IS, IPA, and IAA are associated with changes in gut microbiota composition. Heatmap depicting the Spearman correlation patterns (*P < 0.05, **P < 0.01) of changes in relative abundance of gut bacterial genera and concentrations of blood Trp microbial metabolites. IAA, indole acetate; IPA, indole propionate; IS, indole sulfate; PL, placebo; PomJ, pomegranate juice.

PomJ and PL drink intake did not significantly change α-diversity indices (Chao1 and Shannon; **Supplemental Figure 1**A, B**)**. The β-diversity measure Bray–Curtis dissimilarity was calculated and visualized via PCoA. No distinct separation between baseline and final visits in PL or PomJ groups was observed (Supplemental Figure 1C). Comparing final with baseline in the PL group, the fecal abundance of 3 genera (*Shigella*, *Rothia*, and *Eggerthella*) was increased, and in 2 genera (*Fusobacterium* and *Barnesiella*) it was decreased (*P* < 0.05, **Figure 2**A). Comparing final with baseline microbiota composition in the PomJ group, 3 genera (*Acetitomaculum*, *Faecalicoccus*, and *Kopriimonas*) were increased and 7 genera (*Tyzzerella*, *Turicibacter*, *Parasutterella*, *Catenibacterium*, *Haemophilus*, *Thermotoga*, and *Lactococcus*) were decreased (*P* < 0.05, *P adjusted < 0.2; Figure 2B). Compared with abundance changes between final and baseline in the PL group, 1 y of PomJ intake increased the abundance or reversed the decrease of 2 genera (*Kopriimonas* and *Fusobacterium*), as well as decreased or reversed the increase of 7 genera (*Dorea*, *Catenibacterium*, *Intestinibacter*, *Shigella*, *Parasutterella, Eggerthella*, and *Lactococcus*) (Figure 2C). Some associations were identified between changes in abundance of genera and changes in the Trp microbial metabolites (Figure 2D). For example, the genera *Catenibacterium* and *Sutterrella* were negatively and positively associated with IPA, respectively (Figure 2D).

### Effects of dietary EA and UA supplementation on Trp and its metabolites in mice

**FIGURE 3:**
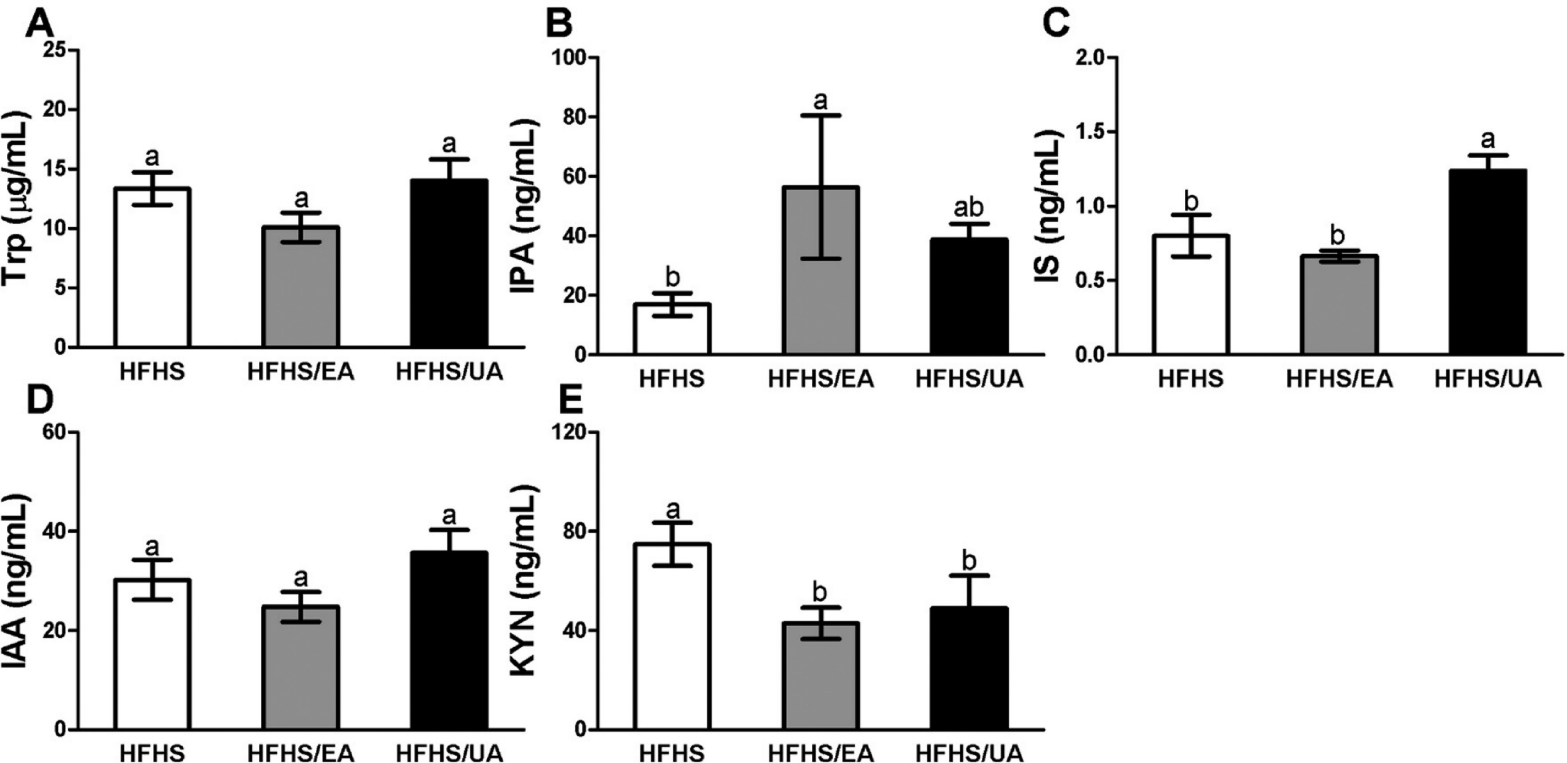
Effects of dietary EA and UA supplementation on serum Trp and its metabolites. (A) Trp, (B) IPA, (C) IS, (D) IAA, and (E) KYN in experimental mice fed for 8 wk with HFHS diet or HFHS diets supplemented with EA or UA. Data are presented as means ± SEMs (HFHS, *n* = 8; HFHS/EA, *n* = 6; HFHS/UA, *n* = 6). Labeled means without a common letter differ, *P* < 0.05. EA, ellagic acid; HFHS, high-fat/high-sucrose; IAA, indole acetate; IPA, indole propionate; IS, indole sulfate; KYN, kynurenine; UA, urolithin A.

**FIGURE 4:**
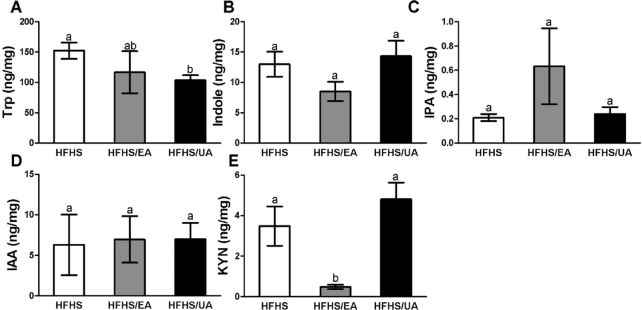
Effects of dietary EA and UA supplementation on cecum Trp and its metabolites. (A) Trp, (B) indole, (C) IPA, (D) IAA, and (E) KYN in experimental mice fed for 8 wk with HFHS diet or HFHS diets supplemented with EA or UA. Data are presented as means ± SEMs (HFHS, *n* = 8; HFHS/EA, *n* = 6; HFHS/UA, *n* = 6). Labeled means without a common letter differ, *P* < 0.05. EA, ellagic acid; HFHS, high-fat/high-sucrose; IAA, indole acetate; IPA, indole propionate; IS, indole sulfate; KYN, kynurenine; UA, urolithin A.

Serum and cecum Trp and its metabolites were evaluated in experimental mice fed an HFHS diet or HFHS diets supplemented with 0.1% EA or UA for 8 wk (**Figures 3** and **4**). In serum, concentrations of the Trp microbial metabolite IPA were significantly increased by EA but not UA, whereas IS was significantly increased by UA but not EA (Figure 3B, C). The KYN pathway is the major route of host-mediated Trp metabolism, and we observed a significant decrease of serum KYN in mice with EA or UA supplementation (Figure 3E). EA and UA did not change serum Trp concentrations (Figure 3A). In cecum content, UA but not EA significantly reduced Trp concentrations, whereas EA but not UA reduced KYN concentrations (Figure 4A, E). Other Trp metabolites with detectable concentrations in cecum, including indole, IPA, and IAA, were not altered by EA or UA (Figure 4B–D).

**FIGURE 5:**
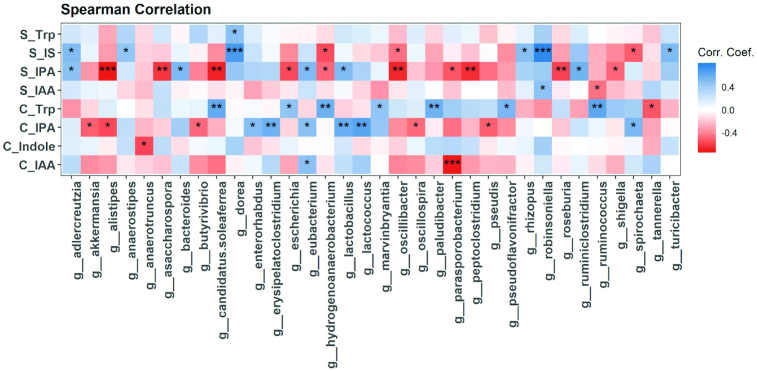
Correlation of cecal genera with cecum and serum Trp and its microbial metabolites. Heatmap depicting the Spearman correlation patterns (*P < 0.05, **P < 0.01, ***P < 0.001) of relative abundance of gut bacterial genera and concentrations of serum (S) and cecum (C) Trp microbial metabolites. IAA, indole acetate; IPA, indole propionate; IS, indole sulfate.

Some associations were identified between relative abundance of cecal genera and Trp and its microbial metabolites in both serum and cecum (**Figure 5**). For example, the abundances of the genera *Lactobacillus* and *Eubacterium* were positively associated with serum and cecum IPA, whereas the abundance of *Alistipes* was negatively associated with serum and cecum IPA (Figure 5).

Other amino acids in serum were not altered by dietary EA or UA supplementation (**Supplemental Figures 2** and **3**).

### Effects of dietary EA and UA supplementation on gut microbiota in mice

**FIGURE 6:**
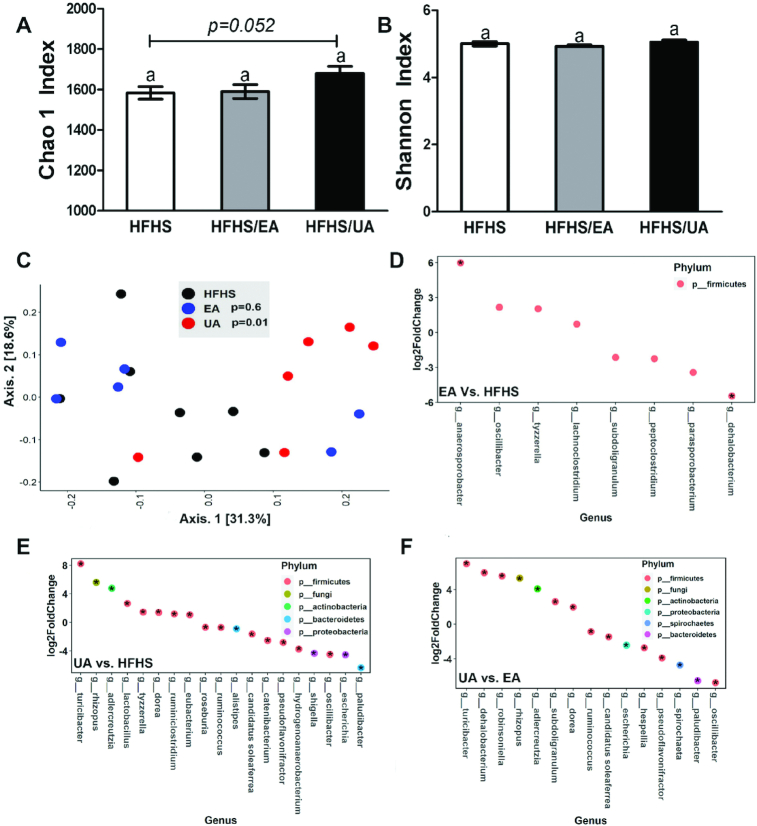
Effects of dietary EA and UA supplementation on gut microbiota. α-Diversity indexes (A) Chao1 and (B) Shannon in experimental mice fed for 8 wk with HFHS diet or HFHS diets supplemented with EA or UA. Data are presented as means ± SEMs (HFHS, *n* = 8; HFHS/EA, *n* = 6; HFHS/UA, *n* = 6). Labeled means without a common letter differ, *P* < 0.05. (C) Principal coordinate analysis plot of β-diversity measure Bray–Curtis dissimilarity. Differential abundance analyses at genus level were performed between (D) EA vs. HFHS, (E) UA vs. HFHS, and (F) UA vs. EA (*P* < 0.05, *adjusted *P* < 0.2). Data plotted as log_2_-fold change. EA, ellagic acid; HFHS, high-fat/high-sucrose; UA, urolithin A.

After 8 wk of dietary supplementation, the mouse gut microbial composition was evaluated using α-diversity indexes including Chao1 for richness and Shannon for richness and evenness combined, as well as β-diversity calculated by Bray–Curtis dissimilarity to evaluate the difference in composition between individual mice (**Figure 6**A–C). UA shaped gut microbiota more potently than EA, as supported by a trend of increase in microbiota richness (*P* = 0.058) as well as a significant difference between clusters when compared with the HFHS group (*P* = 0.01) (Figure 6A, C). Compared with the HFHS diet group, the abundances of 4 genera (*Anaerosporobacter*, *Oscillibacter*, *Tyzzerella*, and *Lachnoclostridium*) were increased and abundances of 4 genera (*Subdoligranulum*, *Peptoclostridium*, *Parasporobacterium*, and *Dehalobacterium*) were decreased by EA (Figure 6D). Compared with the HFHS group, 8 genera (*Turicibacter*, *Rhizopus*, *Adlercreutzia*, *Lactobacillus*, *Tyzzerella*, *Dorea*, *Ruminiclostridium*, and *Eubacterium*) were increased and 11 genera (*Roseburia*, *Ruminococcus*, *Allistipes, Candidatus Soleaferrea*, *Catenibacterium*, *Pseudoflavonifractor*, *Hydrogenoanaerobacterium*, *Shigella*, *Oscillibacter*, *Escherichia*, and *Paludibacter*) were decreased by UA (Figure 6E). Compared with mice fed the EA diet, 7 genera (*Turicibacter*, *Dehalobacterium*, *Robinsoniella*, *Rhizopus*, *Adlercreutzia*, *Subdoligranulum*, and *Dorea*) were higher, and 8 genera (*Ruminococcus*, *Candidatus Soleaferrea*, *Escherichia*, *Hespellia*, *Pseudoflavonifractor*, *Spirochaeta*, *Paludibacter*, and *Oscillibacter*) were lower in the UA group (Figure 6F).

## Discussion

This study investigated the effect of PomJ and its bioactive constituent EA and microbial metabolite UA on Trp metabolism. We first showed that plasma concentrations of the microbial Trp metabolite IPA were maintained in individuals who drank PomJ compared with a decrease observed in the PL group in a small cohort from our recent clinical trial (10). We further demonstrated the effect of EA and UA supplementation on Trp metabolism in a mouse study. Dietary supplementation is a powerful tool in shaping the gut microbial composition as well as microbial metabolism of nutrients. Our data present an example of how phytochemical intake can alter the host and microbial metabolism of nutrients.

UA is a microbial metabolite of ETs/EA, but its potential in regulating microbial composition and metabolic activity has not been documented (26, 27). In this study, we investigated the individual effect of oral supplementation with EA and UA not only on Trp metabolism but also on the microbial composition in DBA/2J mice fed a well-defined HFHS diet and lacking the ability to produce UA. Serum concentrations of IPA and IS were significantly increased by supplementation of EA and UA, respectively (Figure 3). However, IPA concentrations in cecum were similar among experimental groups, and IS concentrations in cecum were below detection limits (data not shown). The KYN pathway in the liver accounts for the majority of dietary Trp degradation (42). The KYN pathway also exists extrahepatically and accounts for minimal Trp degradation, but becomes quantitatively more significant under conditions of immune activation (43). In our mouse study, KYN was reduced by both EA and UA in blood and by EA only in cecum, suggesting the potential effects of EA and UA on the Trp-KYN pathway. Our data overall suggest a potential novel mechanism by which dietary EA and UA affect host physiology by regulating Trp microbial and host metabolism. However, this observation is limited to the mouse model with HFHS-induced IR. Future evaluations of the impact of macronutrient composition as well as host physiological status on EA/UA-mediated Trp metabolism are warranted.

We also evaluated the effects of PomJ intake on fecal microbiota in the small cohort from our recent clinical trial (10). Because the effect of PomJ intake on microbial composition and metabolism was not the primary outcome of this clinical trial, the dietary background of human subjects was not controlled and recorded (10). We observed a large variation when analyzing human gut microbial composition due to the complexity of study participants and the lack of dietary control during the 1-y intervention (Supplemental Figure 1).

The fecal microbial composition was significantly altered in both groups by daily consumption of placebo (sugar-matched) drink or PomJ for 1 y (Figure 2). Changes in the abundance of many genera in human study participants consuming PomJ differed from changes in the mouse microbiota induced by EA or UA supplementation. Our mouse study showed that dietary UA modulated the gut microbiota more potently compared with EA supplementation, as indicated by an increased α-diversity richness index Chao1 as well as significant distinct clustering by β-diversity analysis (Figure 6). In spite of many uncontrolled variables in the human study, 2 genera, *Shigella* and *Catenibacterium*, were found to be decreased by PomJ intake in humans as well as by UA supplementation in mice. *Shigella* is a well-known pathogenic Gram-negative bacterium that causes inflammatory destruction of the intestinal epithelial barrier and has been associated with IBD (44). The exact role of *Catenibacterium* is not well known, but it is positively correlated with the dietary intake of animal fat and is reduced in the gut of colorectal cancer patients (45, 46). In addition, the abundance of *Catenibacterium* and *Shigella* was negatively associated with blood IPA in humans and mice, respectively.

Trp microbial metabolism and its pathways are of great interest and widely explored (1, 6, 47). Recent in silico analysis showed that Trp metabolism pathways that produce neuroactive metabolites are enriched in the 5 genera *Clostridium*, *Burkholderia*, *Streptomyces*, *Pseudomonas*, and *Bacillus* (47). Gut bacteria involved in Trp metabolism include species belonging to *Clostridium*, *Bifidobacterium*, *Lactobacillus*, and *Escherichia* (1, 6). Here we identified a variety of bacterial genera that were positively or negatively correlated with blood and/or cecum Trp metabolites (Figure 2D and Figure 5). For example, *Lactobacillus* was positively correlated with both serum and cecum IPA in mice. Future in vitro and in vivo studies are needed to investigate the cause–effect relation between identified bacterial genera and Trp metabolism, and how EA and UA regulate Trp metabolism.

There are several limitations of the present study. The most important limitation is the small sample size for this subset of the clinical cohort. Due to the small sample size, the between-group difference in the primary cognitive outcome did not reach significance (*P* = 0.27; **Supplemental Table 2**). The second limitation is that PomJ contains a variety of bioactive compounds in addition to ETs and EA, but we only evaluated the impact of EA in the mouse experiment. It would be interesting to study whether other constituents in PomJ also affect Trp metabolism. The third limitation is that although we show the impact of PomJ, EA, and UA on Trp metabolism in human and mice, no analysis of the host and microbial genes or enzymes involved in Trp metabolism was performed. The fourth limitation is that it is unclear how the impact of PomJ, EA, and UA on Trp metabolism subsequently contributes to health benefits of their intake. Additional research is necessary to provide this link.

The present data include analyses of a small cohort of a double-blinded and placebo-controlled trial and mouse feeding study to analyze changes in Trp metabolites in response to consumption of PomJ and EA/UA, respectively. Our data show that dietary PomJ, EA, and/or UA supplementation not only affected the metabolism of the essential amino acid Trp but also altered the gut microbiota composition. In addition, to the best of our knowledge, our data show for the first time that UA is not only a postbiotic, generated from microbial EA metabolism, but also significantly affects microbial composition and metabolism. Manipulation of the complex interplay between diet, microbiota, and host represents a powerful strategy for altering the physiological status of the host.

## Supplementary Material

nzaa165_Supplemental_File

## Notes

This project was supported by the Center for Human Nutrition, Department of Medicine, David Geffen School of Medicine, University of California, Los Angeles.

Author disclosures: GWS owns a US patent (6,274,119) entitled “Methods for Labeling β-Amyloid Plaques and Neurofibrillary Tangles”; Ceremark Pharma, LLC. GWS is among the inventors and is a cofounder of Ceremark Pharma, LLC. He also has served as an advisor to and/or has received lecture fees from AARP, Acadia, Avanir, Genentech, Handok, Herbalife, Medscape, RB Health, Roche, Theravalues, and WebMD; and has received research funds from The Wonderful Company. All other authors report no conflicts of interest.

Supplemental Tables 1 and 2 and Supplemental Figures 1–3 are available from the “Supplementary data” link in the online posting of the article and from the same link in the online table of contents at https://academic.oup.com/cdn/.

Abbreviations used: EA, ellagic acid; ET, ellagitannin; HFHS, high-fat/high-sucrose; IAA, indole acetate; IBD, inflammatory bowel disease; IPA, indole propionate; IR, insulin resistance; IS, indole sulfate; KYN, kynurenine; KYNA, kynurenic acid; LC-ESI-MS/MS, liquid chromatography coupled to electrospray ionization triple quadrupole mass spectrometry; PomJ, pomegranate juice; PCoA, principal coordinate analysis; PL, placebo; UA, urolithin A.
