# Supplementary material for: Pomegranate Metabolites Impact Tryptophan Metabolism in Humans and Mice
Source: Curr Dev Nutr. 2020 Nov 25;4(11):nzaa165. doi: 10.1093/cdn/nzaa165 (PMC7695807; doi:10.1093/cdn/nzaa165)
Supplement: nzaa165_Supplemental_File [file nzaa165_supplemental_file.docx]

**Supplementary Table 1:** Composition of HFHS, HFHS/EA and HFHS/UA diets^1^

| gm% | **HF/HS** | **HF/HS/EA** | **HF/HS/UA** |
| --- | --- | --- | --- |
| Casein | 19.5 | 19.5 | 19.5 |
| DL-Methionine | 0.3 | 0.3 | 0.3 |
| sucrose | 34 | 34 | 34 |
| Corn starch | 15 | 15 | 15 |
| Maltodextrin | 0 | 0 | 0 |
| Milk fat | 21 | 21 | 21 |
| Soybean oil |  | 0 | 0 |
| Cellulose | 5 | 5 | 5 |
| AIN-76 (mineral mix) | 3.5 | 3.5 | 3.5 |
| Vitamin Mix | 1 | 1 | 1 |
| Ethoxyquin | 0.004 | 0.004 | 0.004 |
| Calcium carbonate | 0.4 | 0.4 | 0.4 |
| EA | 0 | 0.1 | 0 |
| UA | 0 | 0 | 0.1 |

^1^HFHS, high-fat/high-sucrose diet; EA, ellagic acid; UA, urolithin A; EA and UA were added to make at 1g polyphenols per 1000g of diet.

**Supplementary Table 2:** BVMT-R Learning Score of study participants

|  | Mean | SD |
| --- | --- | --- |
| PBL | 3.57 | 1.27 |
| PF | 2.57 | 1.27 |
| PJBL | 3.75 | 2.09 |
| PJF | 3.91 | 2.07 |

*PBL placebo baseline; PF placebo final; PJBL PomJ baseline; PJF PomJ final.

*The between-group difference between PomJ and Placebo groups for changes in BVMT-R Learning Score between baseline and 12 month: Mann-Whitney test P = 0.27; within PomJ change: Wilcoxon signed rank test p = 0.91; within PL change: Wilcoxon signed rank test p = 0.31

**Supplementary Figure 1**


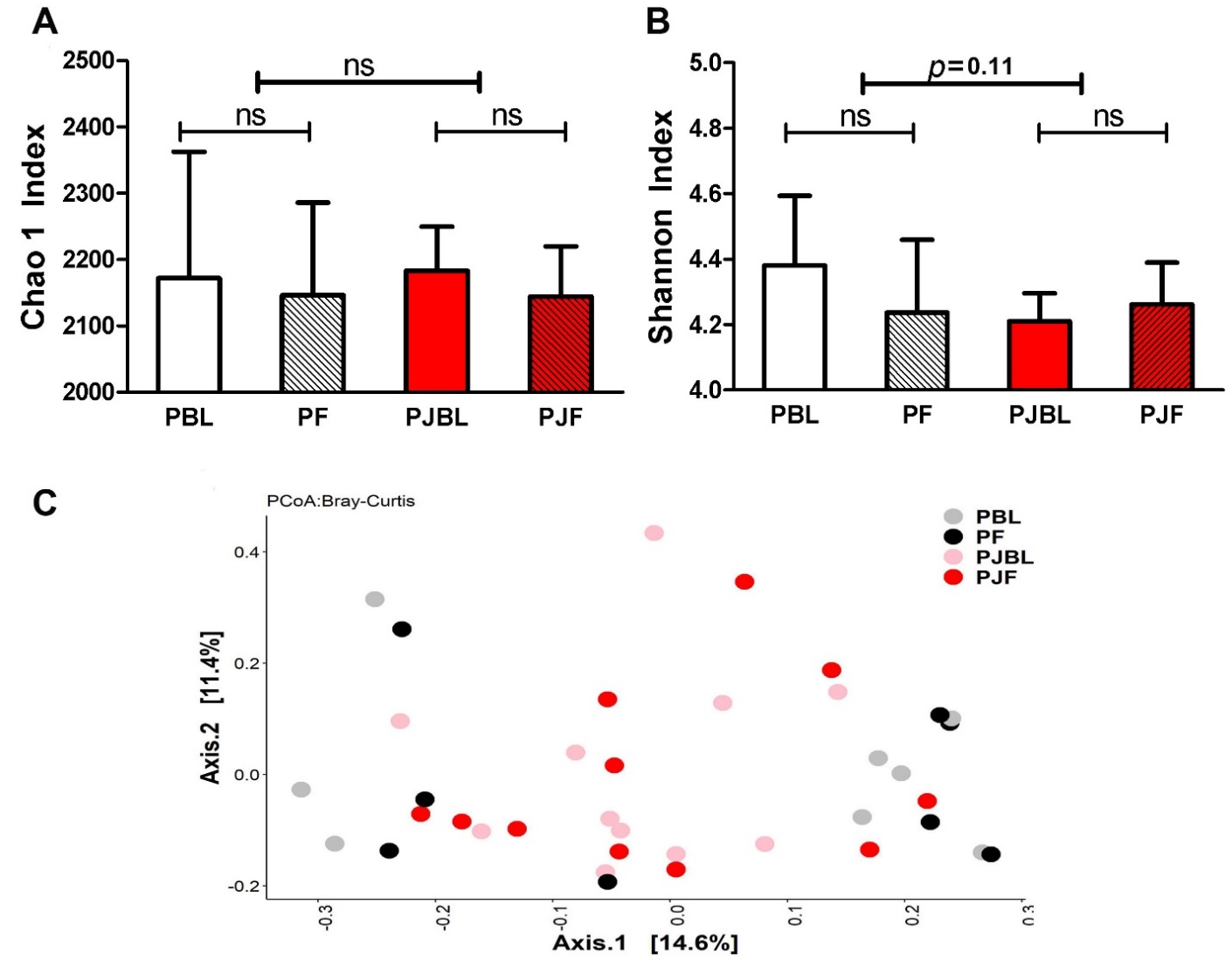


**Supplementary Figure 1**: Microbiome diversity analysis. Alpha diversity analysis of fecal microbiota, Data are displayed as mean ± SEM. (**A**) Chao1 index and (**B**) Shannon index. (**C**) Principal coordinate analysis plot of beta-diversity measure Bray-Curtis dissimilarity. For alpha diversity, ANCOVA model adjusted for baseline value was used to compare over time outcome changes between PL and PomJ groups. Within group changes were analyzed by Wilcoxon signed ranks test**.**

**Supplementary Figure 2**


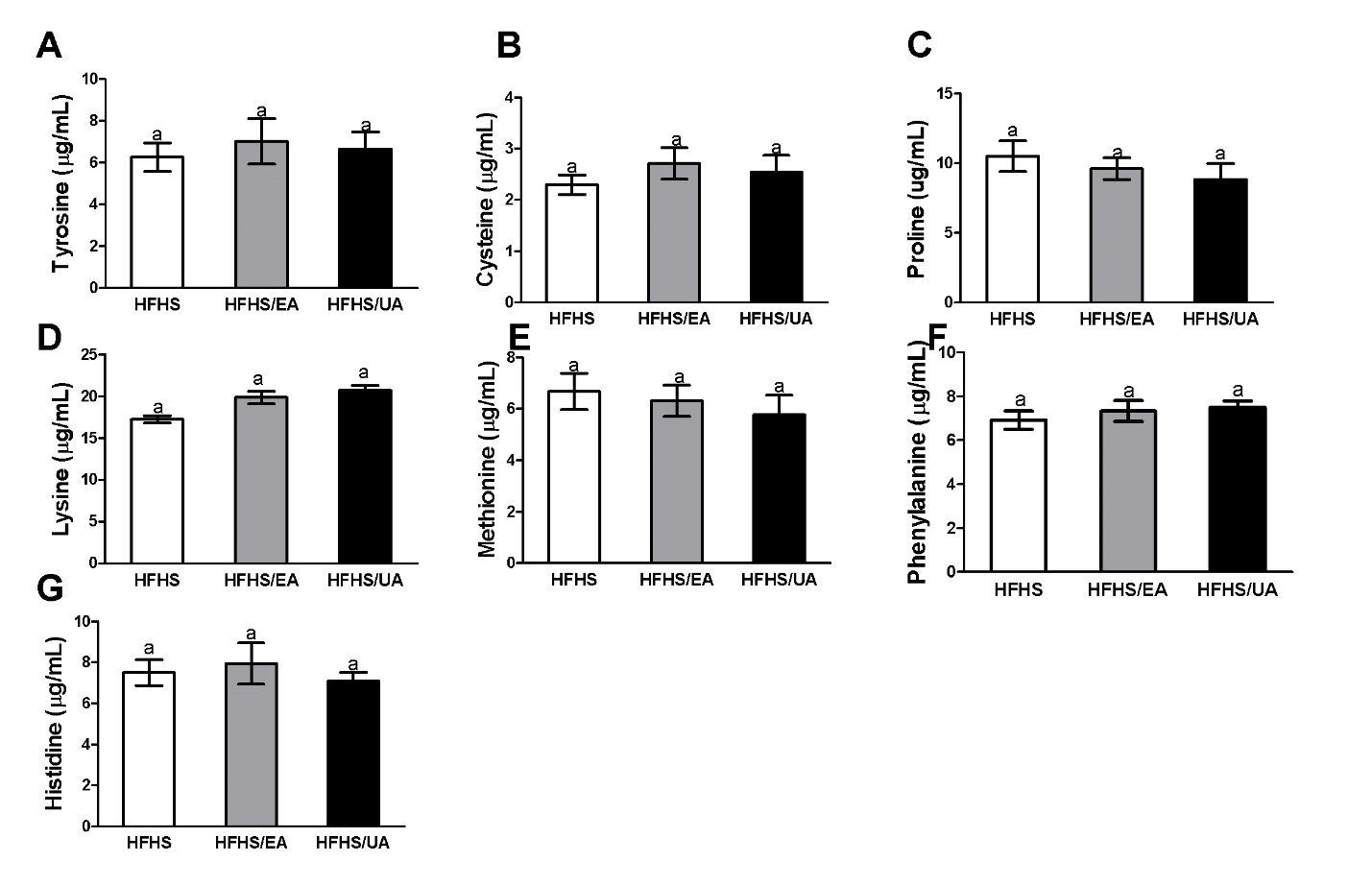


**Supplementary Figure 2:** Serum amino acids in experimental mice fed with HFHS diet or HF/HS diets supplemented with EA or UA for 8 weeks. (A) Tryrosine, (B) Cysteine, (C) Proline, (D) Lysine, (E) Methioinine, (F) Phenylalanine and (G) Histidine. Data are presented as means ± SEMs. Data were analyzed by one-way ANOVA, followed by Tukey-Kramer multiple comparison procedure. Labeled means without a common letter differ, *P*<0.05.

**Supplementary Figure 3**

**
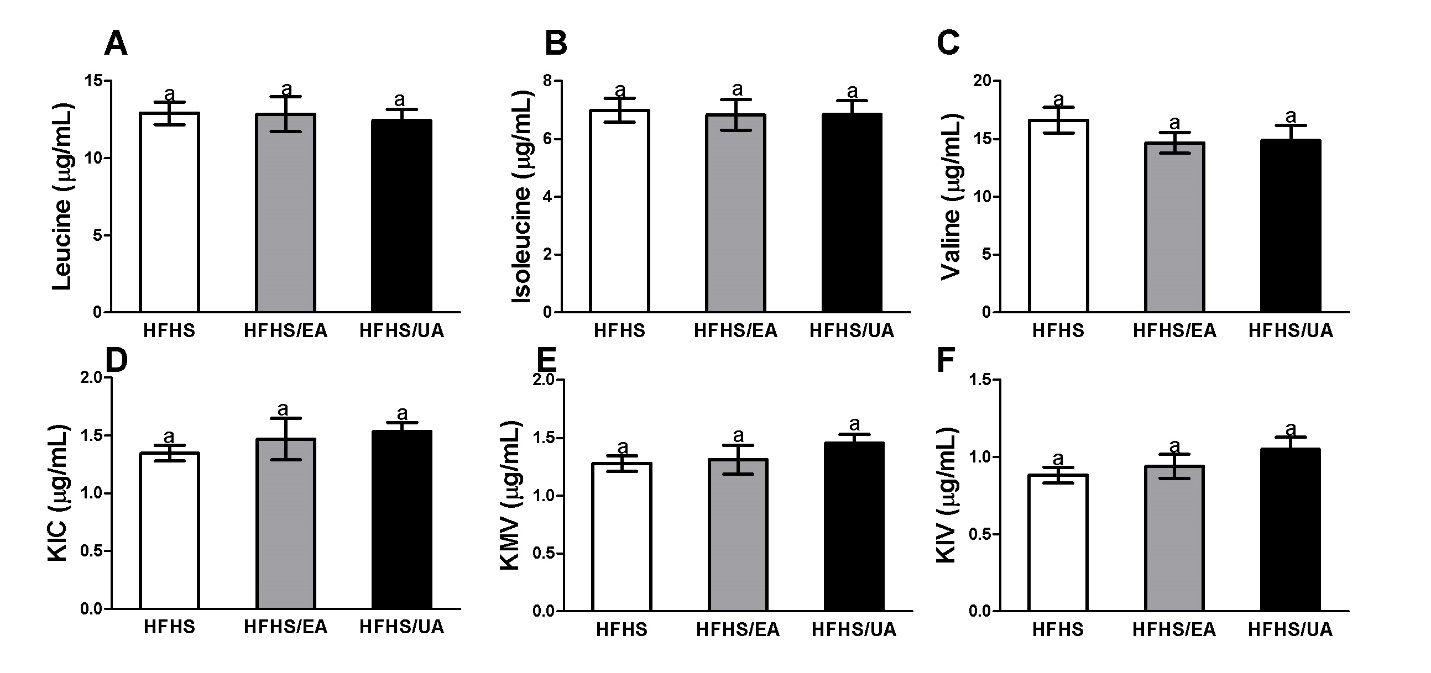
**

**Supplementary Figure 3:** Serum BCAAs and its metabolites BCKAs in experimental mice fed with HF/HS diet or HF/HS diets supplemented with EA or UA for 8 weeks. (A) Leucine, (B) Isoleucine, (C) Valine, (D) alpha-ketoisocaproic (KIC), (E) alpha-keto-beta-methylvaleric (KMV) and (F) alpha-ketoisovaleric (KIV) acids. Data are presented as means ± SEMs. Labeled means without a common letter differ, *P*<0.05.
